# Supplementary material for: TMEM95 is a sperm membrane protein essential for mammalian fertilization
Source: eLife. 2020 Jun 2;9:e53913. doi: 10.7554/eLife.53913 (PMC7295574; doi:10.7554/eLife.53913)
Supplement: Transparent reporting form [file elife-53913-transrepform.docx]

***eLife’s* transparent reporting form**

We encourage authors to provide detailed information *within their submission* to facilitate the interpretation and replication of experiments. Authors can upload supporting documentation to indicate the use of appropriate reporting guidelines for health-related research (see [EQUATOR Network](http://www.equator-network.org/%20)), life science research (see the [BioSharing Information Resource](https://biosharing.org/)), or the [ARRIVE guidelines](http://www.plosbiology.org/article/info:doi/10.1371/journal.pbio.1000412) for reporting work involving animal research. Where applicable, authors should refer to any relevant reporting standards documents in this form.

If you have any questions, please consult our Journal Policies and/or contact us: [editorial@elifesciences.org](mailto:editorial@elifesciences.org).

**Sample-size estimation**

- You should state whether an appropriate sample size was computed when the study was being designed
- You should state the statistical method of sample size computation and any required assumptions
- If no explicit power analysis was used, you should describe how you decided what sample (replicate) size (number) to use

Please outline where this information can be found within the submission (e.g., sections or figure legends), or explain why this information doesn’t apply to your submission:

Sample size was not estimated before-hand because the phenotypic outcome of the intervention (TMEM95 KO) was completely unknown, so we could not estimate an expected variation of the parameters analyzed (related to fertility) to determine a sample size. Given that most fertility parameters analyzed implied animal killing, we kept the samples at the minimum (3-4 animals analyzed per group) to achieve statistical and biological significance.

**Replicates**

- You should report how often each experiment was performed
- You should include a definition of biological versus technical replication
- The data obtained should be provided and sufficient information should be provided to indicate the number of independent biological and/or technical replicates
- If you encountered any outliers, you should describe how these were handled
- Criteria for exclusion/inclusion of data should be clearly stated
- High-throughput sequence data should be uploaded before submission, with a private link for reviewers provided (these are available from both GEO and ArrayExpress)

Please outline where this information can be found within the submission (e.g., sections or figure legends), or explain why this information doesn’t apply to your submission:

The number of biological replicates are indicated below. All data was included (none was excluded). Given the type of parameter analyzed (fertility) technical replicates do not apply in most cases (i.e., there is one measure per biological replicate).

Gene ablation confirmation: Western blot in 3 biological replicates/group (3 sperm samples from 3 different animals per group, Figure 1D) and immunocytochemistry in 3 biological replicates/group (3 sperm samples from 3 different animals per group, Figure 1E).

TMEM95 and IZUMO1 relocalization analysis by immunocytochemistry was performed on 3 biological replicates (3 sperm samples from 3 different animals per group, Figure 1F and figure 1-figure supplement 3)

*In vivo* fertilization. 4 biological replicates/group (4 superovulated females crossed with 4 different males per group, Figure 2A).

*In vitro* fertilization. 3 biological replicates/group (3 IVF assays performed with sperm from 3 different males per group, Figures 2B-C).

Sperm motility analysis: 3 biological replicates/group (3 sperm samples from 3 different animals per group, Figure 2D).

Electron Microscopy analysis: 3 biological replicates/group (3 sperm samples from 3 different animals per group, Figure 2E).

Sperm binding assay: 3 biological replicates/group (3 binding assays performed with sperm from 3 different males per group, 12 oocytes analyzed per male, Figure 2F).

Protein-egg binding assay: 3 replicates/protein tested performed with 10 oocytes/replicating (Figure 2-figure supplement 1).

Sperm penetration (fusion) assay: 3 biological replicates/group (3 fusion assays performed with sperm from 3 different males per group, Figure 2G).

ICSI: 3 biological replicates/group (3 ICSI assays performed on sperm from 3 different males per group, Figure 2H).

Protein interaction (AVEXIS): >3 repetitions using independent protein preparations (Figure 3A).

Cell fusion assay: 3 independent biological replicates (Figure 3B-D; Figure 3-figure supplement 1 C-D´).

**Statistical reporting**

- Statistical analysis methods should be described and justified
- Raw data should be presented in figures whenever informative to do so (typically when N per group is less than 10)
- For each experiment, you should identify the statistical tests used, exact values of N, definitions of center, methods of multiple test correction, and dispersion and precision measures (e.g., mean, median, SD, SEM, confidence intervals; and, for the major substantive results, a measure of effect size (e.g., Pearson's r, Cohen's d)
- Report exact p-values wherever possible alongside the summary statistics and 95% confidence intervals. These should be reported for all key questions and not only when the p-value is less than 0.05.

Please outline where this information can be found within the submission (e.g., sections or figure legends), or explain why this information doesn’t apply to your submission:

Statistical analysis on fertility parameters was analyzed by ANOVA. Differences were considered statistically significant at p<0.001.

(For large datasets, or papers with a very large number of statistical tests, you may upload a single table file with tests, Ns, etc., with reference to sections in the manuscript.)

**Group allocation**

- Indicate how samples were allocated into experimental groups (in the case of clinical studies, please specify allocation to treatment method); if randomization was used, please also state if restricted randomization was applied
- Indicate if masking was used during group allocation, data collection and/or data analysis

Please outline where this information can be found within the submission (e.g., sections or figure legends), or explain why this information doesn’t apply to your submission:

Samples (sperm on the different analyses) were allocated to each experimental group (WT, Hz or KO) based on male genotyping.

**Additional data files (“source data”)**

- We encourage you to upload relevant additional data files, such as numerical data that are represented as a graph in a figure, or as a summary table
- Where provided, these should be in the most useful format, and they can be uploaded as “Source data” files linked to a main figure or table
- Include model definition files including the full list of parameters used
- Include code used for data analysis (e.g., R, MatLab)
- Avoid stating that data files are “available upon request”

Please indicate the figures or tables for which source data files have been provided:

Data are available in the figures, table or as supplementary figures.
